# Shear-Wave Elastography—Diagnostic Value in Children with Chronic Autoimmune Thyroiditis

**DOI:** 10.3390/diagnostics11020248

**Published:** 2021-02-05

**Authors:** Cristina Mihaela Cepeha, Corina Paul, Andreea Borlea, Renata Fofiu, Florin Borcan, Cristina Adriana Dehelean, Viviana Ivan, Dana Stoian

**Affiliations:** 1 PhD School Department, Victor Babes University of Medicine and Pharmacy, 300041 Timisoara, Romania; cristina.cepeha@yahoo.com (C.M.C.); borlea.andreea@umft.ro (A.B.); renata.fofiu@yahoo.com (R.F.); 2 Department of Internal Medicine II, Victor Babes University of Medicine and Pharmacy, 300041 Timisoara, Romania; ivan.viviana@umft.ro (V.I.); stoian.dana@umft.ro (D.S.); 3 Department of Pediatrics, Victor Babes University of Medicine and Pharmacy, 300041 Timisoara, Romania; 4 Faculty of Pharmacy, Victor Babes University of Medicine and Pharmacy, 300041 Timisoara, Romania; fborcan@umft.ro (F.B.); cadehelean@umft.ro (C.A.D.)

**Keywords:** elastography, thyroiditis, thyroid stiffness

## Abstract

Chronic autoimmune thyroiditis (CAT) is the most common thyroid disorder in the pediatric population. Ultrasound evaluation may suggest the diagnosis. Additionally, shear-wave elastography (SWE) proved to be a valuable additional diagnosis tool in adults with CAT by assessing thyroid stiffness (TS). This study aims to assess its use also in detecting children with CAT. The study group consisted of 50 children with confirmed diagnosis of CAT, who were compared to the control group, consisting of 50 children with no thyroid pathology and with an adult group of 50 subjects with CAT. The evaluation included, besides bioimmunochemical evaluation, also thyroid ultrasound evaluation and elastography measurements in the same session (Aixplorer Mach 30, Supersonic imagine, France). The mean TS values were significantly lower for children in the CAT group compared to adults with CAT (15.51 ± 4.76 kPa vs. 20.96 ± 6.31 kPa; *p* < 0.0001) and higher compared to the healthy aged matched controls (15.51 ± 4.76 kPa vs. 10.41 ± 2.01 kPa; *p* < 0.0001). SWE elastography definitely seems a promising technique in the evaluation of children with autoimmune thyroid pathology.

## 1. Introduction

Chronic autoimmune thyroiditis (CAT), subacute thyroiditis (SAT) and Graves’disease (GD) are the major components of diffuse thyroid diseases (DTDs). CAT is characterized by nonspecific chronic inflammation and is defined by the presence of antithyroid antibodies: antithyroid peroxidase antibodies (ATPO) and antithyroglobulin antibodies (ATG) that act against the thyroid tissue causing fibrosis. Studies have shown that CAT is more frequent in females and increases in frequency with age [1,2]. In adult women the incidence reaches up to 27%, while for men it is 7%, increasing after the age of 50 years [3]. Prevalence in children varies between 4 and 10%, being the most common thyroid disorder among pediatric population [4,5]. Hypothyroidism is the most common complication of CAT [6], while the most serious one is the development of thyroid lymphoma [7]. The frequent association of CAT with Turner’s Syndrome and Down’s Syndrome supports the important role of genetic susceptibility of this pathology [8,9].

Ultrasonography (US) is the most widely used imaging method for assessing thyroid morphology, being also easy to use and very well tolerated by the patient. Changes in thyroid volume accompanied by hypoechogenicity, inhomogeneity and pseudonodular appearance may be suggestive for CAT [10,11]. Although DTD can be diagnosed using ultrasound, in daily practice differentiating between types of DTD is difficult and additional diagnostic tools are used.

A new examination technique was recently developed, elastography, a non-invasive method that allows the measurement of tissue elasticity. The two major types of elastography currently used are strain elastography (SE) and shear-wave elastography (SWE). Mulabecirovic et al. compared the performance of different elastography systems and found high intraobserver and interobserver correlation and also good to excellent reproducibility [12]. To quantify tissue elasticity, SE uses a gentle external pressure or an internal physiological pressure (carotid artery pulsation), while SWE uses shock waves generated by the machine in order to measure the tissue stiffness [13,14]. A real-time map is provided by overlapping the SWE image over the image on the B-mode. The operator should not apply any pressure on the probe and the patient is kindly asked to hold the breath for a few seconds. The region of interest (ROI) is placed in the middle of the thyroid and the mean, minimum and maximum stiffness are calculated by the ultrasound software [15,16]. There are currently numerous studies that have shown the usefulness of elastography in differentiating malignant thyroid lesions from benign ones with excellent results [17,18].The usefulness of thyroid elastography in the evaluation of diffuse thyroid pathology was also studied [19,20], but at the present there are insufficient data regarding the performance of elastography in the examination of children and adolescents diagnosed with CAT.

Therefore, the aim of this paper is to evaluate the performance of SWE in the diagnosis of CAT in children and to make a comparative evaluation of the degree of fibrosis between children and adults.

## 2. Materials and Methods

### 2.1. Subjects

One hundred and fifty subjects were evaluated; 100 of them were children/adolescents and 50 adults. Out of the 100 children included, 50 of them were diagnosed with CAT, the other half representing the control group without thyroid pathology. The two groups of children were age and gender matched. All the adults included were also diagnosed with CAT.

### 2.2. Inclusion Criteria

We included children aged 5–18 years old, diagnosed with CAT. The diagnosis was based on clinical examination and ultrasound appearance. The diagnosis was confirmed by immunology assays: high levels of ATPO and/or ATG antibodies. The control group included 50 children (age 5–18) without thyroid pathology. The adult group consisted of 50 subjects over the age of 20 diagnosed with CAT by the same means of examination.

### 2.3. Exclusion Criteria

We excluded from our study both adults and children diagnosed with Graves’disease (GD) or presenting nodular thyroid pathology or malignancies. We also excluded cases with a history of thyroid surgery (lobectomy/thyroidectomy). Adults without thyroid pathology were not included either. Additionally, children under the age of 5 were not included in the study due to difficult examination. Cases with normal antithyroid antibodies titers but suggestive ultrasound aspect for CAT were not taken into consideration.

### 2.4. Biochemical Assay

We took into consideration the following measurements: free-thyroxine (FT4) (reference range 0.93–1.7 ng/dL; method immunochemistry with enzyme chemiluminescence immunoassay—ECLIA), thyroid-stimulating hormone (TSH) (reference range 0.27–4.20 μUI/mL; method ECLIA), ATPO (reference range <34 UI/mL for adults; <26 UI/mL for children; method microparticle-based chemiluminescence immunochemistry –CMIA) and ATG (reference range <115 UI/mL for adults; <64 UI/mL for children, method ECLIA). All biochemical test were performed in an accredited laboratory.

### 2.5. Conventional Ultrasound and Elastography Examination

**Figure 1:**
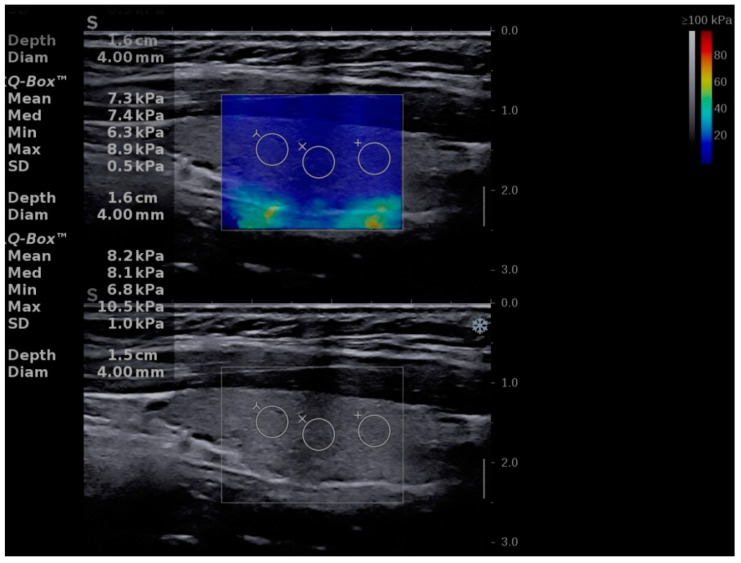
Shear-wave elastography of a healthy child, boy, 13 years old.

**Figure 2:**
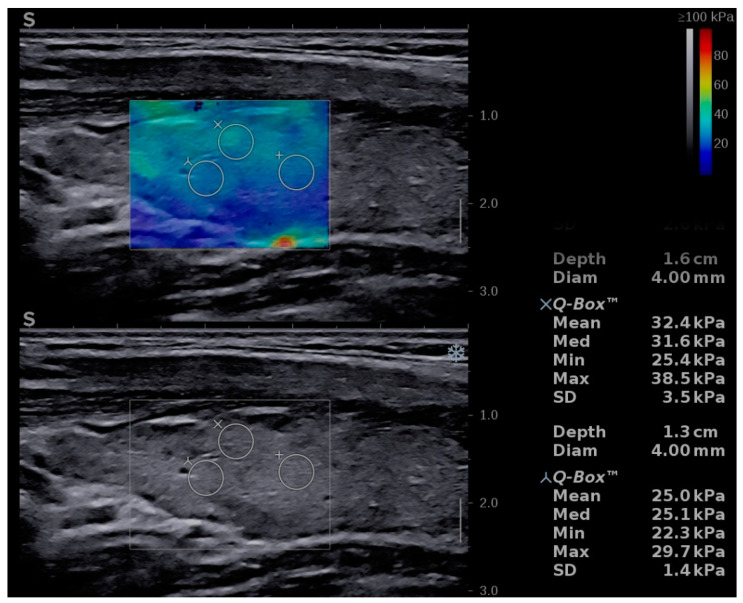
Shear-wave elastography of a girl, 16 years old—from the chronic autoimmune thyroiditis (CAT) group.

**Figure 3:**
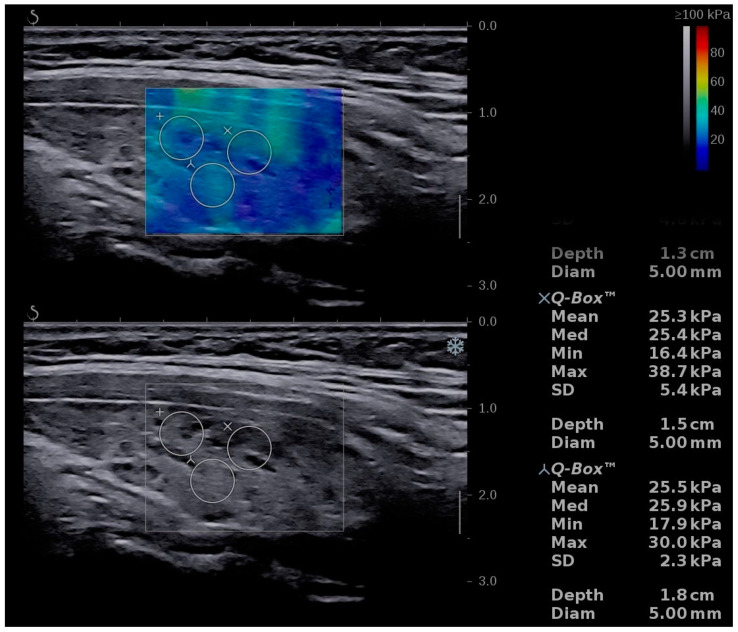
Shear-wave elastography of an adult patient with CAT, 36 years old, female.

The Aixplorer Mach 30 (Supersonic imagine, Aix-en-Provence, France) machine, with an L 18-5 probe (linear, 5–18 MHz), was used to perform conventional B-mode thyroid ultrasound followed by shear-wave elastography. The examinations were performed by two experienced sonographers. The subject was placed in supine position with the neck fully exposed. A grey-scale ultrasound was performed and for each lobe three diameters were measured. The thyroid volume was displayed and expressed in milliliters (mL) as recommended [21]. After conventional US, SWE was performed during the same visit. The probe was placed on one side of the neck and SWE mode was initiated. The machine provides a color map from blue (indicating soft tissue) to red (indicating hard tissue). All images were obtained in the longitudinal plane (Figure 1, Figure 2 and Figure 3). The subject was asked to hold his/her breath for about five seconds. Consequently, the image was frozen and the tissue elasticity was measured using the Q-BOX and recorded as kilopascals. The region of interest (ROI) was placed approximately in the middle of the thyroid lobe. The ROI was placed in hypoechoic regions, if any of these were detected. Six measurements were performed for each subject. A quantitative elasticity value was expressed in kilo-Pascals (kPa).

### 2.6. Statistical Analysis

The statistical analysis was performed using MedCalc Statistical Software version 12.5.0.0 (MedCalc Software bv, Ostend, Belgium) and SPSS Statistics for Windows, Version 17.0. (SPSS Inc., Chicago, IL, USA) The Kolmogorov–Smirnov test was used for testing the distribution of numerical variables. Numerical variables with normal distribution were presented as the mean value and standard deviation, while variables with non-normal distribution were presented as median values and range intervals. Categorical variables were presented as the number (proportion) of patients with/without the specific characteristic.

The Student’s *t*-test was used for group comparisons of continuous variables with a normal distribution and nonparametric tests (Mann Whitney U test) were used for variables with non-normal distribution. Group comparisons of categorical variables were performed using Pearson’s χ^2^-test. The Pearson correlation coefficient (r) was used to established correlations between thyroid stiffness (TS) and others parameters (age and ATPO levels).

Univariate regression analysis and multivariate regression analysis were used in order to identify the independent predictors of CAT. Areas under receiver operating characteristic curves (AUROCs) were determined for TS values to identify discriminating cut-offs. The optimal cut-off values were determined from AUROC curve analysis and the Youden J index and its associated criterion values were chosen. Positive predictive value (PPV—true positive cases/all positive cases), negative predictive value (NPV—true negative cases/all negative cases) and diagnostic accuracy (sum of true positive and true negative cases/total number of cases) were calculated. Ninety-five percent confidence intervals were determined for each predictive test and a *p*-value < 0.05 was considered to reveal statistical significance.

## 3. Results

Reliable thyroid stiffness (TS) measurements using 2D-SWE were obtained in 100/100 children (100%), 50 diagnosed with CAT, 50 aged matched without thyroid pathology, respectively, and in 50/50 (100%) adults diagnosed with CAT, therefore 150 subjects were included in the final analysis. The main characteristics of the subjects included are summarized in Table 1.

For each child, 2D-SWE was performed on both thyroid lobes and no differences were found between the mean values obtained in the left lobe and the right lobe, respectively (15.47 ± 4.77 kPa vs. 15.56 ± 5.22 kPa; *p* = 0.92). The mean thyroid stiffness value for children with CAT was 15.51 ± 4.76 kPa.

The same approach was used for adults and no differences were found between the mean values obtained in the left lobe and the right lobe, respectively (21.17 ± 6.13 kPa vs. 20.76 ± 7.07 kPa; *p* = 0.75). The mean TS value for adults with CAT was 20.96 ± 6.31 kPa.

The mean TS values were significantly lower for children in the CAT group compared to adults with CAT (15.51 ± 4.76 kPa vs. 20.96 ± 6.31 kPa; *p* < 0.0001). Additionally, in children, the mean TS values were significantly higher compared to the healthy aged matched controls (15.51 ± 4.76 kPa vs. 10.41 ± 2.01 kPa; *p* < 0.0001) (Table 2).

The thyroid volume was higher in children with CAT compared to those without (13.69 ± 7.1 mL (2.04–39.5) vs. 9.6 ± 3.4 mL (3.2–16.2), *p* = 0.004), while in adults, the mean values for thyroid volume were 13.58 ± 5.6 mL (4.25–37.92).

In children with CAT we did not observe any difference between the mean TS related to gender (15.73 ± 3.31 kPa vs. 15.48 ± 4.93 kPa; *p* = 0.9128).

**Figure 4:**
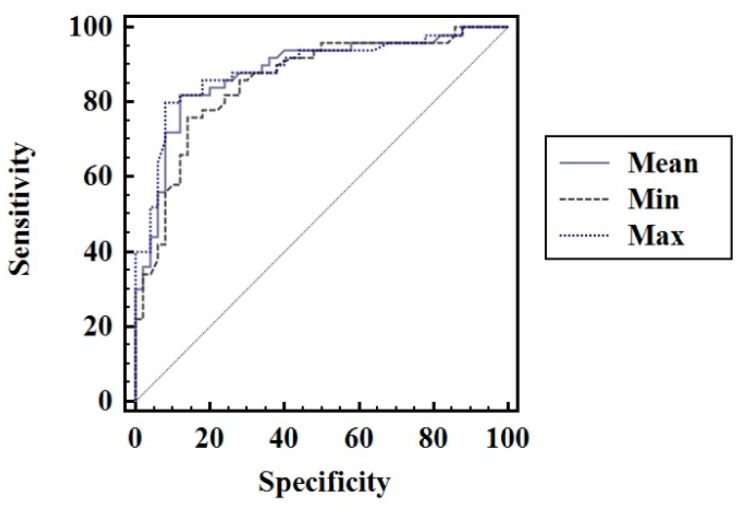
Comparison between receiver operating characteristic for TS values, using mean TS values, the highest TS values and the lowest TS values.

The optimal cut-off value defined as the highest sum of sensitivity(Se) and specificity (Sp) determined using the mean TS values for predicting the presence of CAT in children was >12.2 kPa (AUROC—0.88, Se—82%, Sp—88%, PPV—87.2% and NPV—83%). In the same population, when the highest TS values were used in the analysis, the following cut-off value for predicting CAT: TS >13.13 kPa (AUROC—0.89, Se—80%, Sp—92%, PPV—91% and NPV—82.1%) was obtained; when the lowest TS values were used in the analysis, the following cut-off value was obtained: TS > 11.53 kPa (AUROC = 0.85, Se—76%, Sp—86%, PPV—84.4% and NPV—78.2%) (Figure 4). The difference between their prediction accuracies is summarized in Table 3.

As observed in Figure 4, the highest AUROC was observed for TS Max values, followed by TS Mean, respectively, and TS Min values.

In the univariate analysis we observed a significant difference between patients with or without CAT in the following parameters: thyroid stiffness values, ATPO antibodies levels and ATG antibodies levels (Table 4).

Age, thyroid stiffness values, ATPO antibodies levels, ATG antibodies levels, TSH levels and FT4 levels were tested using univariate regression analysis, as independent predictors for CAT. Thyroid stiffness values (*p* < 0.0001), ATPO antibodies levels (*p* < 0.0001) and ATG antibodies levels (*p* < 0.0001) were independent predictors of CAT in children.

In multivariate logistic regression the model including TS values, ATPO antibodies levels and ATG antibodies levels showed that all the parameters were independent predictors for CAT: TS values (β = 0.046 ± 0.009, *p* < 0.0001), ATPO antibodies levels (β = −0.00045 ± 0.00008, *p* < 0.0001) and ATG antibodies levels (β= −0.00018 ± 0.000072, *p* = 0.01). The following regression equation was determined: 0.046 × TS − 0.00045 × ATPO − 0.0015 × ATG and TS had the highest relative contribution in this prediction model (β = 0.046).

A weak correlation was found between ATPO levels and TS values (r = 0.43) and also between TS values and age (r = 0.30). No correlation was found between Ts values and ATG level, TSH, FT4 or thyroid volume.

**Figure 5:**
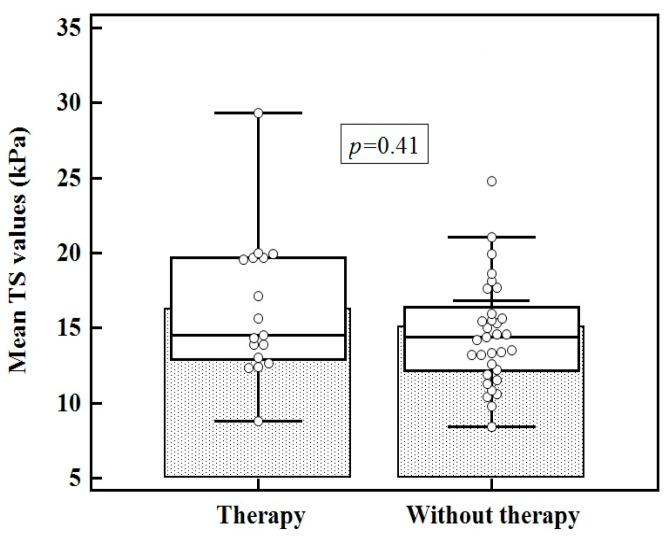
Comparison between children in CAT group, with and without therapy.

Of the children in the CAT group 17/50 (34%) were receiving LT4replacementtherapy. No differences were found between the mean TS values in children undergoing therapy compared to the TS in those without therapy (16.29 ± 4.75 kPa vs. 15.11 ± 4.79 kPa; *p* = 0.41) (Figure 5).

## 4. Discussion

Being one of the most common endocrine pathologies, CAT has been studied in detail over the years. Numerous factors can influence the ultrasound appearance of the thyroid, including obesity and lymphocytic infiltration [22]. Thus, it is known that the histological substrate is represented by lymphocytic infiltration, the firm consistency of the thyroid being given by the different degrees of fibrosis. Inhomogeneity results from the different degrees of thyrocyte lesions [23].

Given that inflammation increases the level of fibrosis resulting in increased inelasticity, it is worth verifying the inverse hypothesis: whether an increased level of fibrosis is describing the presence of inflammation.

A study conducted on 107 healthy children evaluated the elasticity scores and proposed the median value 6.38 ± 1.97 kPa as the standard value for non-pathological thyroid [24]. Our analysis found higher values in healthy children, the mean TS = 10.41 ± 2.01 kPa. Another study compared the mean values of elasticity in CAT and control group found the mean TS for control group TS = 16.18 ± 5.4 kPa [25]. As there is so much variability, studies on larger groups of children are needed. Additionally, the size of the ROI and its depth could be considered in the evaluation. The tissue elasticity in children was also studied using SE. The mean strain index value of normal thyroid gland was 0.54 ± 0.38 [26].

According to the statistical analysis, a cut-off value of 12.2 kPa for the mean SWE value was established in this study for predicting CAT, with a sensitivity of 82% and specificity of 88%. For the maximum SWE, a cut-off value 13.13 kPa discriminated between CAT and normal subjects with a sensitivity of 80% and specificity of 92%. Similar results were obtained in a study that included 59 pediatric patients diagnosed with CAT and 26 healthy volunteers. The cut-off value with the highest diagnostic accuracy for elasticity values was 12.3 kPa (Se 86.4, Sp 96.3%) [27]. Differences between thyroid elasticity levels of healthy children and children diagnosed with CAT were also observed using strain elastography. The strain index (SI) was found to be significantly higher in children with CAT compared to healthy children (1.75 ± 1.46 vs. 0.26 ± 0.77; *p* < 0.001) [28].

We found no correlation between thyroid elasticity and TSH values, in accordance with other studies’ findings [29,30], only a weak correlation between ATPO levels and thyroid elasticity. Similarly, one study showed a moderate positive correlation between TS and ATPO (r = 0.40; *p* < 0.05) but no correlation between TS and ATG [27], while in another study, a weak positive correlation was found between TS and ATG (r = 0.343; *p* = 0.004) and no correlation between TS and ATPO [31].

An interesting remark is the correlation between TS and age of subjects (r = 0.30). Similar results were obtained in one study that evaluated elasticity scores of the thyroid, submandibular and parotid glands. The mean elasticity values for the thyroid were 14.6 ± 3.3 kPa and a significant correlation noted was between age and TS value (r = 0.38) [32]. A significant positive correlation between TS and age was also found in one study conducted in Turkey (r = 0.390, *p* < 0.001) [29].

No differences in TS values were found between girls and boys, in accordance with the literature [29,30].

We also studied whether there is any difference between the TS in children diagnosed with CAT who are on hormone replacement therapy with levothyroxine and those who are not. We did not find significant differences in elasticity between the two subgroups of pediatric patients. A study conducted by Magri showed significant differences between the thyroid elasticity of patients undergoing treatment and those without treatment (27.3 ± 9.0 kPa vs. 20.9 ± 10.4 kPa; *p* = 0.02). It should be mentioned that the subjects of this study were adults [33]. Similar results were noted using SE (3.45 ± 2.53 vs. 2.15 ± 1.27; *p* < 0.0001) [19]. We consider it important that in pediatric patients no significant differences were found between the two subgroups. Studies on larger groups of children are necessary to verify whether the observed results in adults can be applied in children.

The relatively small number of subjects included wasthe limitation of the study, but the difference in the prevalence of CAT between adults and children must also be taken into consideration.

A very important aspect is that the values obtained in the group of children diagnosed with CAT were compared with two control groups: one group of healthy children and another group of adults diagnosed with CAT. To our knowledge, this aspect has not been studied comparatively so far. We aim to follow the evolution of these patients over a longer period of time and to study the usefulness of elastography in determining the progression of the disease.

## 5. Conclusions

The prevalence of CAT in the pediatric population is considerable, presenting with euthyroidism in most of the cases. Thyroid ultrasound suggests the diagnosis in most of the cases, and elastography was proven to be able to make a distinction between healthy and CAT subjects in the adult population. This study raises important questions regarding the use of elastography also for detecting CAT in the pediatric population, with good results so far. Another important finding relates to the significant differences noted between adults with CAT and children with the same condition. The study supports the use elastography, when available, for the evaluation of children with suspected of autoimmune thyroiditis.

## Figures and Tables

**Table 1:** Main characteristics of the study group (*N*—number of subjects).

Parameter	Children with CAT	Healthy Children	*p* Value	Adults with CAT
*N*	50	50		50
Age (median value and rage interval)	13.5 (5–18)	13.5 (5–18)		43.02 (24–72)
Gender (%):				
Male	5/50 (10%)	5/50 (10%)		2/50 (4%)
Female	45/50 (90%)	45/50 (90%)		48/50 (96%)
Thyroid volume (Mean ± SD)	13.69 ± 7.10	9.6 ± 3.47	0.004	13.59 ± 5.62
Levothyroxine (LT4) replacement therapy	17/50 (34%)	0/50		15/50 (30%)
No treatment	33/50 (66%)	50/50		35/50 (70%)

**Table 2:** Mean thyroid stiffness values in the two children groups (values are expressed in kPa ± SD).

Parameter	Children with CAT	Healthy Children	*p* Value
Mean TS values	15.51 ± 4.76	10.41 ± 2.01	*p* < 0.0001
Left lobe mean values	15.46 ± 4.77	10.32 ± 2.22	*p* < 0.0001
Right lobe mean values	15.56 ± 5.22	10.50 ± 2.14	*p* < 0.0001

TS—thyroid stiffness; CAT—chronic autoimmune thyroiditis.

**Table 3:** The difference between areas under receiver operating characteristic curves (AUROCs) for the established cut-off value.

Parameter	Mean vs. Min	Mean vs. Max	Min vs. Max
AUROC	0.882 vs. 0.857	0.882 vs. 0.890	0.857 vs. 0.890
Difference between areas	0.0254	0.00800	0.0334
Standard Error	0.0141	0.0103	0.0226
95% CI	−0.00225 to 0.0531	−0.0122 to 0.0282	−0.0109 to 0.0777
z statistic	1.801	0.777	1.479
*p* value	*p* = 0.0718	*p* = 0.4369	*p* = 0.1392

**Table 4:** Comparison between TS values and laboratory parameters in children with CAT vs. healthy children.

Parameter	Children with CAT *n* = 50	Healthy Children *n* = 50	*p*
TS values	15.51 ± 4.76	10.41 ± 2.01	<0.0001
ATPO	646.2 ± 512.6	12.06 ± 6.3	<0.0001
ATG	388.17 ± 659.27	13.09 ± 5.3	0.0001
TSH	3.94 ± 3.49	3.12 ± 1.68	0.13
FT4	1.15 ± 0.26	1.16 ± 0.22	0.83

TS—thyroid stiffness; CAT—chronic autoimmune thyroiditis; ATPO—antithyroid peroxidase antibodies; ATG—antithyroglobulin antibodies; TSH—thyroid-stimulating hormone; FT4—free-thyroxine.

## Data Availability

The data presented in this study are available on request from the corresponding author. The data are not publicly available due to patient privacy IRB requirement.
